# Solution to travelling salesman problem by clusters and a modified multi-restart iterated local search metaheuristic

**DOI:** 10.1371/journal.pone.0201868

**Published:** 2018-08-22

**Authors:** Gustavo Erick Anaya Fuentes, Eva Selene Hernández Gress, Juan Carlos Seck Tuoh Mora, Joselito Medina Marín

**Affiliations:** Engineering Academic Area, Autonomous University of Hidalgo, Pachuca, Hidalgo, Mexico; Universidad Nacional de Mar del Plata, ARGENTINA

## Abstract

This article finds feasible solutions to the travelling salesman problem, obtaining the route with the shortest distance to visit *n* cities just once, returning to the starting city. The problem addressed is clustering the cities, then using the *NEH* heuristic, which provides an initial solution that is refined using a modification of the metaheuristic Multi-Restart Iterated Local Search *MRSILS*; finally, clusters are joined to end the route with the minimum distance to the travelling salesman problem. The contribution of this research is the use of the metaheuristic *MRSILS*, that in our knowledge had not been used to solve the travelling salesman problem using clusters. The main objective of this article is to demonstrate that the proposed algorithm is more efficient than Genetic Algorithms when clusters are used. To demonstrate the above, both algorithms are compared with some cases taken from the literature, also a comparison with the best-known results is done. In addition, statistical studies are made in the same conditions to demonstrate this fact. Our method obtains better results in all the 10 cases compared.

## 1 Introduction

Travelling Salesman Problem *TSP* is well known in the literature and is considered one of the most difficult problems to solve, besides being very useful to solve various problems in manufacturing. The first time who someone tried to solve this problem was addressed by Dantzig, Fulkerson and Johnson [1] algorithm on an IBM 7090 computer, the method used was Branch and Bound, through this method it was found that the average computational time was too high to be feasible to solve. Since then, *TSP* has been solved by various Metaheuristics such as Ant Colony *ACO*, Simulated Annealing *RS*, Genetic Algorithms *GA*, among others, but new algorithms continue to emerge, and it is interesting proven them in classic problems.

All the methods used to solve TSP have found a limit on their computational runtime, we attemting to solve problems with many cities or nodes [2], because this problem is NP Hard [3]. For this reason, the *TSP* remains a subject of current research to try new and different heuristic strategies. There are different applications in problems with a lot of nodes. For example, the Family Travel Salesman Problem, that is motivated by the order picking problem in warehouses where products of the same type are stored in different warehouses or in separate places in the same warehouse [4]. Other application of the TSP is in the technical approach to solve the fuel optimization problem in separated spacecraft interferometry missions [5]. Also, different problems can be converted to TSP with a lot of nodes, one of them is the Vehicle Routing Problem [6], and other is the Job Shop Scheduling Problem [7]; in the the last case a problem with 30 jobs and 10 machines is a TSP with 300 cities. Other applications are in Tas, Gendreau, Jabali and Laporte [8] and in Veenstra, Roodbergen, Vis and Coelho [9]. In a different topic, different clustering techniques have been used to solve problems with many nodes, such as clusters based in prototypes, centers, graphs and densities [10]. Some authors have already solved the *TSP* by clusters, see for example the work of Phienthrakul [11], what hence forth we will named as *CTSP* (Clustering the Traveling Salesman Problem). In this research, he solved the problem with Ant Colony, Simulated Annealing and Genetic Algorithms., but the best results that he obtained were with Genetic Algorithms.

Our proposal is the solution of *CTSP* applying a combination of heuristics as the *NEH* and a modification of the metaheuristic Multi Restart Iterated Local Search *MRSILS* [12], all these terms together will be named as *CTSPMRILS* (The Travelling Salesman Problem with Clusters, *NE*H and Multi Restart Iteration Local Search). Until today, no one who has solved it in this way has been found, and this is the innovative part. The approach in this paper is tested in 10 instances of Phienthrakul [11]. The *CTSPMRILS* finds satisfactory results in all the instances proved. The aim of this article is to demonstrate that the proposed algorithm *CTSPMRILS* is more efficient than Genetic Algorithms when clusters are used.

This article is structured as follows: section 2 shows the *TSP* background, the clustering techniques and their application in the TSP, and also some basic aspects related to the *NEH* heuristic and *MRSILS*; section 3 presents the description and problem statement, where defines the problem solving in mathematical terms; section 4 describes the development of the proposed algorithm in this article; later in section 5 the results are presented; in section 6 a discussion of the results is provided. Finally, section 7 presents the conclusions of this research.

## 2 Background

### 2.1 The travelling salesman problem

The TSP can be formally defined as follows (Buthainah, 2008). Let a network *G =* [*N*,*A*,*C*], that is *N* the set nodes, *A* the set of arcs, and *C* = [*c*_*ij*_] the cost matrix. That is, the cost of the trip since node *i* to node *j*. The TSP requires a Halmiltonian cycle in *G* of minimum cost, being a Hamiltonian cycle, one that passes to through each node *i* exactly once. TSP is a problem of permutation that aims to find the path of shorter length or minimum cost in an unguided graph than represents the cities or nodes to be visited. The TSP starts in a node, visiting all the nodes one by one to finally return to the initial node, in such a way must form routes and no sub-paths. The TSP can be modeled through Integer Programming [13] and in the symmetric case, Branch and Cut algorithms have been developed. Although the search for optimal solutions of large instances of the symmetric TSP via Branch and Cut have been reached, this effort is two-fold; one must invest in a relevant algorithmic and implementation effort. The implementation effort is unfortunately now far too high for a newcomer [14]. TSP is considered NP-complete and is one of the biggest challenges faced by analysts, even through various techniques that are available [15].

To deal with the complexity of the problem, TSP has been studied extensively with meta heuristics, see for example, the works of Dorigo [16] with colony of ants, Cerny [17] with the Monte Carlo Method; Jog et al. [18], Chattarjee et al. [19], Larrañaga et al.[20], Moon et al. [21], Fogel [22], Also, different versions of *GA* have been presented in Kurian, Mathew and Kumar [15] intended to improve efficiency in solving the *TSP*, so far without finding a method or technique that ensures finding the optimum in polynomial time. Current trends to solve TSP problems includes the Clustering Technique or solve the *TSP* separately generating smaller problems as described in the next section.

### 2.2 Clustering techniques

Arising from the difficulties in finding solutions for the *TSP* in feasible time, works such as Dutta and Bhattacharya [23] discusses various techniques of clustering based on policies and methods of clusters, they show the steps for the clustering process and discuss some important concepts related to class data and the characteristics of selection and evolution of the cluster, which is a term that has its beginnings in Amdahl's Law [24]. In addition, the results found by Dutta and Bhattacharya [23] indicate that clustering techniques can be classified into 7 groups: based on distances, densities, models, on pictures, in seeds, spectra and hierarchies used in data mining. Clustering has been used to solve different problems applied in different fields, for example Nizam [25], proposed clustering as a powerful control system voltage stability and presents a new technique for clustering called neural Kohonen network. The formation of these clusters can simplify the control voltage. Vijayalakshmi, Jayanavithraa, Ramya [26] observed in the field of genetics that are measured levels of thousands of genes simultaneously, using microarray technology. In this technology, genetic clusters approach is used to find genes with similar functions. Under this approach, several clustering algorithms are used in clusters; as proposed by Vijayalakshmi et al. [26], which is an automatic algorithm that provides the ability to find a strong global convergence towards an optimal solution.

Weiya, Guohui and Dan [27], proposed a novel method called cluster graph consistent approach, the solution obtained by this method is close to the optimal with a discrete solution. The different techniques of clustering are also analyzed for data mining by authors such as Saroj and Chaudhary [28]. Clusters group is a subject of active research in many fields such as statistics, identifying patterns and learning machines. Cluster analysis is an excellent tool to work with a lot of data.

Moreover, Kaur and Kaur [29] uses clustering in Data Mining by *k-*means clustering to divide the data into *k* clusters; Besides, Nadana and Shriram [30] proposed a methodology called Megadata based on a model of clustering for large data sets. The experimental results showed that it is possible to find a better quality of clusters without improving the computational time.

Kaur and Singh [31] proposed an advanced clustering algorithm to direct large data sets. This advanced method for clustering allows to measure the distance of each object, also requires a simple data structure for each iteration. Their experimental results proved that the advanced method of clustering algorithm can improve the effectiveness of the speed and accuracy of the algorithm by reducing the computational complexity.

Tavse and Khandelwal [32] classified data internet clusters for application in data transmission, achieving better efficiency, longer life and stability of the network, optimizing data classification. Refianti et al [33] compared two algorithms called: *affinity propagation* and *k*-means, both grouped data clusters. The data are regarding the timing of completion of the thesis students. The results show that the *k*-means algorithm provides more accurate results with cluster data and more effectively than *affinity propagation*, while this provides different values for the centroids after five tests. In the next section, clustering to find better solutions to the *TSP* is presented.

### 2.3 Clusters applied to the travelling salesman problem

Different methods and techniques have been used to solve the *TSP* clustered, as Lin-Kernighan proposed by Karapetyan and Gutin [34]. Also, the *GA* with clusters *CA*G presented recently at work Sivaraj, Ravichandran and Devipriya [35], who notes that using *CAG* manages to find the optimal solution in less time that standard *GA* named *SGA*, this was observed in three cases shown in Sivaraj et al. [35]. The latter author developed an unsupervised learning mechanism, used to group similar objects in clusters, ensuring that despite the different techniques for clustering that are available, there is a general strategy that works in the same way on different problems. However, the conclusion is that it is better to use simple mechanisms.

In the origins of the clusters Tsai and Chiu [36] proposed a very similar to *CTSP* method called hierarchical clustering, which adopts an ambitious strategy to gradually mix objects and build a classification structure called dendrogram. Nevertheless, the quality of its clusters is unreliable. To overcome the problem, a global optimum strategy for the construction of the dendrogram is to find the optimal circular route that minimizes the total distance to visit all objects along the arms of the dendrogram, which is modeled as a *TSP* and is solved using a method of search variable in the neighborhood. When the cluster dendrogram is modeled, it is based on information provided by the order. Through these experiments, the quality of this clustering method is superior to traditional methods.

Nagy and Negru [37] discussed methods to cluster which can be used to treat spatial and temporal patterns in a large amount of data. They use 55 cities to apply the methods of detection. His approach allows us to observe the existence of different spatial and temporal clusters.

Vishnupriya and Sagayaraj [38] implemented clustering algorithms for techniques used in data mining, making possible the analysis of data sets, using the algorithm *k-*means to calculate the value of the cost based on the Euclidean distance like *TSP*.

Nidhi [39] proposes the *k-*means algorithm for the problem of increasing data with several clusters generated dynamically and without repetition, which reduces the computational time, providing more accurate results. Therefore, the initial grouping is done with statistical data, using *k*-means. Then the next points, the largest distance between the centroid and the farthest point is used to define the next point that is in the cluster, repeating the process to cover the total data.

Derived from the works mentioned above, it becomes necessary to define a heuristic that may help to solve the *TSP* with feasible results, hence, in this article the use of *NEH* and *MRSILS* algorithms is proposed as a feasible alternative.

### 2.4 NEH y multi-restart iterated local search

Nawas, Enscore and Ham [40] proposed a heuristic called *NEH* which intends to solve the Job Shop Scheduling Problem, Liu Song and Wu [41] improved this algorithm with two techniques. First, to reduce the computational time per block properties are developed and introduced in the *NEH* algorithm to obtain a shorter the computational time. Second, tiebreaker rules are applied to obtain good solutions. The simulation results show that these two techniques improve the results obtained in the *NEH* Algorithm.

Mestría [42] also proposed a heuristic method to solve the *CTSP*, which it is a generalization of *TSP* where a set of nodes is divided into disjointed clusters with the aim of finding the minimum cost of the Hamiltonian cycle. Mestría, [42] developed two random descendants in the neighborhood, with iterated local search called ILS algorithm to solve the *CTSP*. The computational time obtained shows that the heuristic methods are competitive using software in parallel.

Grasas, Juan and Lorenzo [43] found that *ILS* is one of the most popular solutions using simple heuristics. *ILS* is recognized by many authors as relatively simple as well as having a structure capable of dealing with combinatorial optimization problems *COPs*. The *ILS* has been successfully applied to provide near optimal solutions for different problems of logistics, transportation, production etc. However, it has been designed to solve problems in deterministic scenarios, therefore, it does not reflect the actual stochastic nature of the systems.

Dong, Chen, Huang and Nowak [44] proposed the *MRSILS* to solved Flow Shop Scheduling Problem, *MRSILS* generates an initial solution as well as constructs in negligible time and the corresponding *ILS* performs. This is repeated until a termination criterion, it can be set as the maximum number of iterations for the local search procedure or the maximum allowable computational time.

Seck et al. [12] modifies the *MRSILS* algorithm with an uncomplicated process which generates minor changes by means of permutations for improving the initial solution before using *MRSILS*, then a minor variation is made in the *MRSILS* to obtain better performance. The experiments show that the new algorithms produce slightly better results than the original one.

Thus, it is proposed to try *MRSILS* and *NEH* heuristic to apply on clusters of the problem described below.

## 3 Description and problem statement

The TSP can be defined as follows: Find the shortest route for a sales person starting from a city, visiting each in a specific group of cities just once and returning to the starting point [45].

The TSP can be defined as an undirected graph *G* = (*V*,*E*) if symmetric, or as a direct graph *G* = (*V*,*A*) if it is asymmetric. The set *V* = {1,…*n*} is a set of vertices or nodes, *E =* (*i*,*j*): *i*, *jϵV*, *i<j* a set of arches undirected, *A* = {(*i*,*j*): *i*, *jϵV*, *i≠j*} a set of directed arcs. *A* is the cost matrix *C = C*_*ij*_ defined on *E* or possibly on *A*. The cost of the matrix satisfies the triangle inequality [46] provided *C*_*ij*_ *≤ C*_*ik*_*+C*_*kj*_, for all *i*,*j*.*k*; where vertices are points in in the plane *P*_*i*_ = (*X*_*i*_,*Y*_*i*_); and $C_{ij}=\sqrt{{\left(X_{i}-X_{j}\right)}^{2}+{\left(Y_{i}-Y_{j}\right)}^{2}}$ is the Euclidean distance. The triangle inequality is satisfied if *C*_*ij*_ is the length of the shortest path from *i* to *j* in *G*.

Anil, Bramel and Hertz [47] defines the *CTSP* considering ordering the clusters for *TSP*, where a traveling salesman starts and ends its journey in a specific city must visit a set of *n* points divided into *k* clusters not connected, the *k* points of that cluster are visited before the points of the cluster *k+*1 for *k =* 1,2,…,*k–1* seeking the minimum total travel distance.

Given a complete undirected graph *G* = (*V*,*E*) where *k+*1 clusters denoted by *C*_*i*_ ⊆ *V*, for each *i* = 0,1,2,…,*k*, preestablished. It is assumed that *C*_*i*_ *∩ C*_*j*_ *=* 0 for all 1≤*i*, *j≤k*, *i≠j*, and *C*_0_ is denoted as a single node 0_ϵ_*V* and may be a deposit *C*_0_ = 0. The *CTSP* seeks to determine the minimum distance of commuter travel agent starting and ending in the same city and visiting each of them, which are referred to as *V* and are in one way. To solve this problem, Phienthrakul [11] proposed a technique called *k*-means, to group in clusters with the steps described below:

1. Choose an integer value for *k*.
2. Select *k* objects arbitrarily (use these as initial set of *k* centroids).
3. Assign each of the objects to a cluster, which is closest to the centroid.
4. Recalculate the centroid of *k* clusters.
5. Repeat steps 3 and 4 until the centroids do not change more.

Another technique proposed by the author is called Gaussian Mixed Model applied by the normal distribution forming clusters. The model uses the Maximization Algorithm *Hope EM* [48], to adjust the Gaussian distribution of the data. The algorithm starts by defining the number of clusters *k* and selecting the settings *k* of Gaussian distributions

*λ* = (*μ*_1_,*μ*_*2*_,…,*μk*,*σ*1,*σ2*,…,*σk*) where each cluster has a normal probability distribution with N $\left(\mu_{i},\sigma_{i}{}^{2}\right)$.

This article proposes to use *k*-means algorithm and recalculate the centroids by deducting the arithmetic mean of the coordinates *X* and *Y*, to obtain a new centroid and iterate until the centroids no change more, allowing the algorithm to be more efficient by using the arithmetic mean instead of a fit test that requires more steps.

## 4 Development

This article seeks to solve the *TSP* in combination with clusters, *NEH* and *MRSILS*, such combination henceforth it is called as *CTSPMRSILS*, which consist in grouping nodes in clusters to find the minimum distance in each of them, but unlike the proposed by Phienthrakul [11] it is modified to work with a proposed heuristic that provides solutions for each cluster with a combination of the *NEH* [49] and MR*SILS* algorithms, which is explained by applying it to the instance *burma*14 instance of *TSPLIB* [50], as shown in the following steps:

1. Let *n* the number of cities or nodes to visit by the commercial traveler, the number of groups or clusters in which the total of nodes is divided, calculating *k*. So that $k=\sqrt{\frac{n}{2}}$, rounding the value of *k* when necessary. To illustrate the solution to the problem, coordinates *X* and *Y* are taken from *burma*14 [50], which are shown in Table 1; for this example, $k=\sqrt{\frac{14}{2}}\approx 3.$

**Table 1:** Coordinates instance burma14, TSPLIB [50].

	Coordinates X	Coordinates Y
1	16.47	96.10
2	16.47	94.44
3	20.09	92.52
4	22.39	93.37
5	25.23	97.24
6	22.00	96.05
7	20.47	97.02
8	17.20	96.29
9	16.30	97.38
10	14.05	98.12
11	16.53	97.38
12	21.52	95.59
13	19.41	97.13
14	20.09	94.55

2. *k* clusters are represented individually by nodes called centroids placed at random coordinates on the *TSP*; in this case, *k* random numbers are the centroids in *X* between the minimum value of the coordinates 14.05 and 25.23 as maximum; similarly, the random number in *Y* is between the minimum 92.54 and maximum 98.12. Thus, the centroid *N* = (*Coordinate X*, *Coordinate Y*) is obtained for *N* = 1,2,*k*. And the following centroids are generated:
  Centroid 1 = (22, 98);
  Centroid 2 = (18, 97);
  Centroid 3 = (23, 9).
3. Subsequently, *n* nodes are grouped by assigning each of them to the nearest centroid, such that no node remains without assigned centroid; as it is shown in Table 2 for this example.

**Table 2:** Clusters allocation by the minimum distance from the node to the centroid.

Node	Centroid 1	Centroid 2	Centroid 3	Cluster
1	5.85	1.78	7.23	2
2	6.58	2.98	6.69	2
3	5.78	4.93	2.95	3
4	4.65	5.70	0.71	3
5	3.32	7.23	4.79	1
6	1.95	4.11	3.21	1
7	1.82	2.47	4.75	1
8	5.10	1.07	6.67	2
9	5.73	1.74	8.00	2
10	7.95	4.11	10.31	2
11	5.51	1.52	7.81	2
12	2.46	3.79	2.98	1
13	2.73	1.42	5.47	2
14	3.94	3.22	3.30	2

Table 2 also shows the distance between each node and each cluster; each node is assigned to the nearest cluster, using the expression of the distance between two points Franklin [51]:

$$d=\sqrt{{\left(X_{1}-X_{2}\right)}^{2}+{\left(Y_{1}-Y_{2}\right)}^{2}}$$ (1)

The assignment of the nodes to the clusters is as follows, cluster *1 = 5*, *6*, *7*, *12*; cluster *2 = 1*, *2*, *8*, *9*, *10*, *11*, *13*, *14* and cluster *3 = 3*, *4*.

4. Then, the arithmetic mean of the coordinates is calculated in *X* and *Y* for each cluster, with the intention of finding a representative node in each of them; these nodes replace the centroids of step 3. Steps 3 and 4 are repeated until the centroids no change more. For example, *burma14* [50] centroids are updated as:
  Centroid 1 = (22.31,96.48);
  Centroid 2 = (17.07, 96.42);
  Centroid 3 = (21.24,92.96).

Table 3 shows the coordinates of clusters 1,2 and 3; *C1*, *C2*,*C3* respectively in the *X* and *Y* axes, also from it the average of the coordinates of each cluster and in both axis are obtained to recalculate the centroids, therefore, such averages are: for *C*_1_,*μx* = 22.31 and *μγ* = 96.48; for *C*_2_,*μx* = 17.07 and *μγ* = 96.42; for *C*_3_,*μx* = 21.24 and *μγ* = 92.96.

**Table 3:** Reallocation of centroids.

CIX	CIY	C2X	C2Y	C3X	C3Y
25.23	97.24	16.47	96.10	20.09	92.54
22.00	96.05	16.47	94.44	22.39	93.37
20.47	97.02	17.20	96.29		
21.52	95.59	16.30	97.38		
		14.05	98.12		
		16.53	97.38		
		19.41	97.13		
		20.09	94.55		

Repeating steps 3 and 4, Table 4 is obtained; in which there is only one modification compared to Table 3. The clusters are remapped as shown in Table 4.

**Table 4:** Reallocation of centroids.

CIX	CIY	C2X	C2Y	C3X	C3Y
25.23	97.24	16.47	96.10	20.09	92.54
22.00	96.05	16.47	94.44	22.39	93.37
20.47	97.02	17.20	96.29	20.09	94.55
21.52	95.59	16.30	97.38		
		14.05	98.12		
		16.53	97.38		
		19.41	97.13		

Now new centroids are:

Centroid 1 = (22.31, 96.48);
Centroid 2 = (16.63, 96.69);
Centroid 3 = (20.85, 93.48);

For the next iteration, there is no reassignment of nodes to a different cluster, allocations are equal to those of Table 4, so the step 4 in this iteration found the following clusters:

Cluster 1 = 5-6-7-12;5
Cluster 2 = 1-2-8-9-10-11-13;
Cluster 3 = 3-4-14.
  5. In this step the NEH algorithm is applied to each of the *k* clusters, hereinafter the algorithm only be explained with the cluster 2, which is the largest for this example, calculating the cost or distance of each of the nodes to the other nodes belonging to the cluster in Table 5.

**Table 5:** NEH start cost cluster 2.

Node	1	2	8	9	10	11	13	Cost
1	0.0	1.6	0.7	1.2	3.1	1.2	3.1	11.2
2	1.6	0.0	1.9	2.9	4.4	2.9	3.9	17.9
8	0.7	1.9	0.0	1.4	3.6	1.2	2.3	11.4
9	1.2	2.9	1.4	0.0	2.3	0.2	3.1	11.3
10	3.1	4.4	3.6	2.3	0.0	2.5	5.4	21.6
11	1.2	2.9	1.2	0.2	2.5	0.0	2.8	11.2
13	3.1	3.9	2.3	3.1	5.4	2.8	0.0	20.9

6. Nodes are sorted in ascending order relative to the travel expense, thereby Table 6 for Cluster 2 is obtained. Chosen as initial cluster nodes in question in the order obtained in the previous step with what you have for each cluster route.

**Table 6:** NEH initial nodes sortered for cluster 2 output nodes.

Output nodes	Cost
11.00	11.21
1.00	11.25
9.00	11.37
8.00	11.44
2.00	17.92
13.00	20.93
10.00	21.61

This order of the nodes is:

Centroid 1 = (22.31, 96.48);
Centroid 2 = (17.07, 96.42);
Centroid 3 = (21.24, 92.96)
  7. In this step the first two nodes of the list are chosen to exchange them and the permutation that provides the minimum cost is chosen, this is shown in Table 7 for cluster 2.

**Table 7:** Permutation 11–1, cluster2.

Permutations	Cost
11–1	1.2814
1–11	1.2814

This permutation is joined by the third node 9, moving its position in the list, Table 8 is obtained.

**Table 8:** permutations 9-11-1, cluster2.

Permutations	Cost
9-11-1	1.5114
11-1-9	1.5212
11-1-9	2.5726

The best result obtained in Table 9 can be considered as 9-11-8-1; and it is incorporated to node 2, for Table 10.

**Table 9:** Permutation 8-9-11-1, cluster2.

Permutation	Cost
8-9-11-1	2.9249
9-8-11-1	3.9744
9-11-8-1	2.2638
9-11-1-8	2.2657

**Table 10:** Permutation 2-9-11-8-1, cluster2.

Permutation	Cost
2-9-11-8-1	5.2087
9-2-11-8-1	7.9193
9-11-2-8-1	5.9138
9-11-8-2-1	5.1583
9-11-8-1-2	3.9238

The best result is that follows the path 9-11-8-1-2, to which node 13 is incorporated as shown in Table 11. The smaller travel distance in Table 11, is the path 13-9-11-8-1-2, so that the end node 10 of this cluster permuting as shown in Table 12.

**Table 11:** Permutation 13-9-11-8-1-2, cluster2.

Permutation	Cost
13-9-11-8-1-2	7.0438
9-13-11-8-1-2	9.7046
9-11-13-8-1-2	7.8994
9-11-8-13-1-2	8.6489
9-11-8-1-13-2	9.3639
9-11-8-1-2-13	7.9087

**Table 12:** Permutation 10-13-9-11-8-1-2, cluster2.

Permutation	Cost
10-13-9-11-8-1-2	12.4945
13-10-8-9-11-1-2	13.6786
13-8-10-9-11-1-2	11.5472
13-8-9-10-11-1-2	11.6758
13-8-9-11-10-1-2	11.4081
13-8-9-11-1-10-2	12.8459
13-8-9-11-1-2-10	11.3536

The results in each cluster by *NEH* algorithm are: cluster1, *π*´ = 7 − 12 − 6–5 with cost of 5.8812; cluster2, *π* = 13−8−9−11−1−2−10 with cost of 11.3536 and cluster3, *π*´ = 4−3−14 with cost of 4.4552.

8. The next step is to apply the *MRSILS* algorithm to each of the clusters starting from the initial solution generated by the *NEH* and arbitrarily define the number of iterations of the procedure that are performed, as well as a provisional store *π* with a default capacity number *nπ*, which stores the value of *π* at the end of each iteration. In this initial solution, *π*, each of the nodes are moved, respecting the order in which they appear, changing their position and choosing the one with the lowest cost or maintaining the one already stored, if it has a lower cost. To see the example of the 13th node, refer to the Table 13. The initial solution provided by NEH for Cluster 2 was 13-8-9-11-1-2-10 and then node 8 is showed in Table 14. In which the value of *π* with *π*´ = 13−8−9−11−1−2−10 and we observe that *π*´ = *π*, such that it does not change its value.

**Table 13:** Permutation node 13 in cluster2.

Permutation	Cost
13-8-9-11-1-2-10	11.3536
8-13-9-11-1-2-10	13.0601
8-9-13-11-1-2-10	14.7702
8-9-11-13-1-2-10	13.7140
8-9-11-1-13-2-10	14.4295
8-9-11-1-2-13-10	14.0205
8-9-11-1-2-10-13	14.4400

**Table 14:** Permutation node 8 in cluster2.

Permutation	Cost
8-13-9-11-1-2-10	13.0601
13-8-9-11-1-2-10	11.3536
13-9-8-11-1-2-10	13.1588
13-9-11-8-1-2-10	11.4482
13-9-11-1-8-2-10	11.7790
13-9-11-1-2-8-10	11.9232
13-9-11-1-2-8-10	14.3388

Then the next node 9 moves position. See Table 15, in this case *π*´ = 13−8−11−9−1−2−10 is updated because it has a lower cost compared with *π*. Each of the remaining nodes is changed into its position, the results being as follows. For the 11th node π'=13−8−11−9−1−2−10, for node 1, *π*´ = 13−8−9−11−1−2−10, the change of node 2 keeps *π*´ constant. Finally, for node 10, *π*´ = 13−8−9−11−10−1−2.

**Table 15:** Permutation node 9 in cluster2.

Permutation	Cost
9-13-8-11-1-2-10	14.1096
13-9-8-11-1-2-10	13.1588
13-8-9-11-1-2-10	11.3536
13-8-11-9-1-2-10	11.2294
13-8-11-1-9-2-10	13.5657
13-8-11-1-2-9-10	11.8986
13-8-11-1-2-9-10	13.3581

The procedure is repeated in each cluster to a predetermined number of iterations. The value of *π* in each cluster is stored in the stack named *π* with capacity *nπ* in each cluster. When the number of iterations exceeds the value of *nπ*, the worst of the values in *π* will be eliminated from the iteration *nπ*+1. In addition, when a new iteration is initiated a perturbation is made on the best value of *π* by generating two random positions to make a shift. These are called *Aleat* and *Aleat*2 using this new individual generated as *π* for start the next iteration of MRSILS. For example, if the best element of *π* is 13-8-11-9-1-2-10, *Aleat*1 = 3 and *Aleat*2 = 6. The perturbed solution is 13-8-10-11-9-1-2. In this example, only one iteration is perfomed, and the metaheuristic *MRSILS* is concluded. Now the clusters are: *Cluster1*, *π* = 7−12−6−5 with cost of 5.8812; Cluster2, *π* = 13−8−11−9−1−2−10 with cost of 11.2294 and Cluster3, *π* = 4−3−14 with cost of 4.4552.

9. Now, with this routes for each cluster, a procedure is performed to obtain a single route; for this, initially the centroid 1 distance is calculated for each of the remain centroids, in order to identify which cluster is closest to cluster 1; and this cluster is called cluster near $C_{C}.$ For *burma*14, the distance from centroid 1 = (22.31, 96.48) is calculated, to each of the rest of the centroids, using the Eq (1) whereby Table 16 is obtained; it is observed that centroid 3 is closest to centroid 1, and then it is called cluster near C_c_.

**Table 16:** Distance from centroid 1 to each centroid.

	Centroid 2	Centroid 3
**Centroid 1**	5.67611663	3.33640525

10. The distance between centroid 1 and each of the nodes in *C*_*c*_ are calculated, and the closest node to centroid 1 is chosen; it is named as node*C2*. The distances between centroid 2 and each of the nodes or cluster 1 are calculated, also the nearest node to centroid 2 is chosen and named as node *C1*. Subsequently, Cluster 1 is joined with *C*_*c*_, through the node*C1* and node*C2*. The distances between Centroid1 and each of the nodes *C*_*c*_., where the centroid 1 coordinates are *X* = 22.31 and *Y* = 96.48, are shown in Table 17.

**Table 17:** Distance from C_c_ to each node.

Node	X	Y	Distance to Centrode 1
14	20.09	94.55	2.94
3	20.09	92.54	4.52
4	22.39	93.37	3.11

Table 17 shows the closed node to centroid 1 is 14, so node C2 = 14. The distance of C_c_ with coordinates X = 20.85 and Y = 93.48 is calculated for each node of cluster 1, as shown in Table 18. That can find the node of cluster 1 and it is closer to the centroid 2, in this case corresponds to node 12 so node C1 = 12.

**Table 18:** Distance from centroid 2 to cluster 1 nodes.

Node	X	Y	Distance to centroid 2
7	20.47	97.02	3.56
12	21.52	95.59	2.21
6	22	96.05	2.81
5	25.23	97.24	5.77

**Table 19:** Distance node 14 to close nodes (3,4).

Node	Distance to node 14
3	2.01
4	2.59

11. Now, it is checked which of the nodes attached to node *C2* of *C*_*c*_, is located closer to it, thereby choosing the direction of travel within the cluster. The last node of the path in *C*_*c*_, is called *ClusterEnd*2, remaining free according to the algorithm until joining the last cluster with it. Similarly, the last node in cluster 1 is called ClusterEnd1. For the example, the distance of *nodeC*2 = 14, to respect node 3 and node 4, is calculated. Table 19 shows such distances. So, node 3 is the closest, the direction the C_c_ route must follow as 14-3-4; in addition,*ClusterEnd*2 = 4

To end the direction of travel for cluster 1, it is necessary to end the nearest node to node node C1 = 12 between 6 and 7; which are the only possible consecutive as obtained in for its respective Cluster, as shown in Table 20.

**Table 20:** Distance node 12 to close nodes (6,7).

Node	Distance to node 12
6	0.6648
7	1.7741

**Fig 1:**
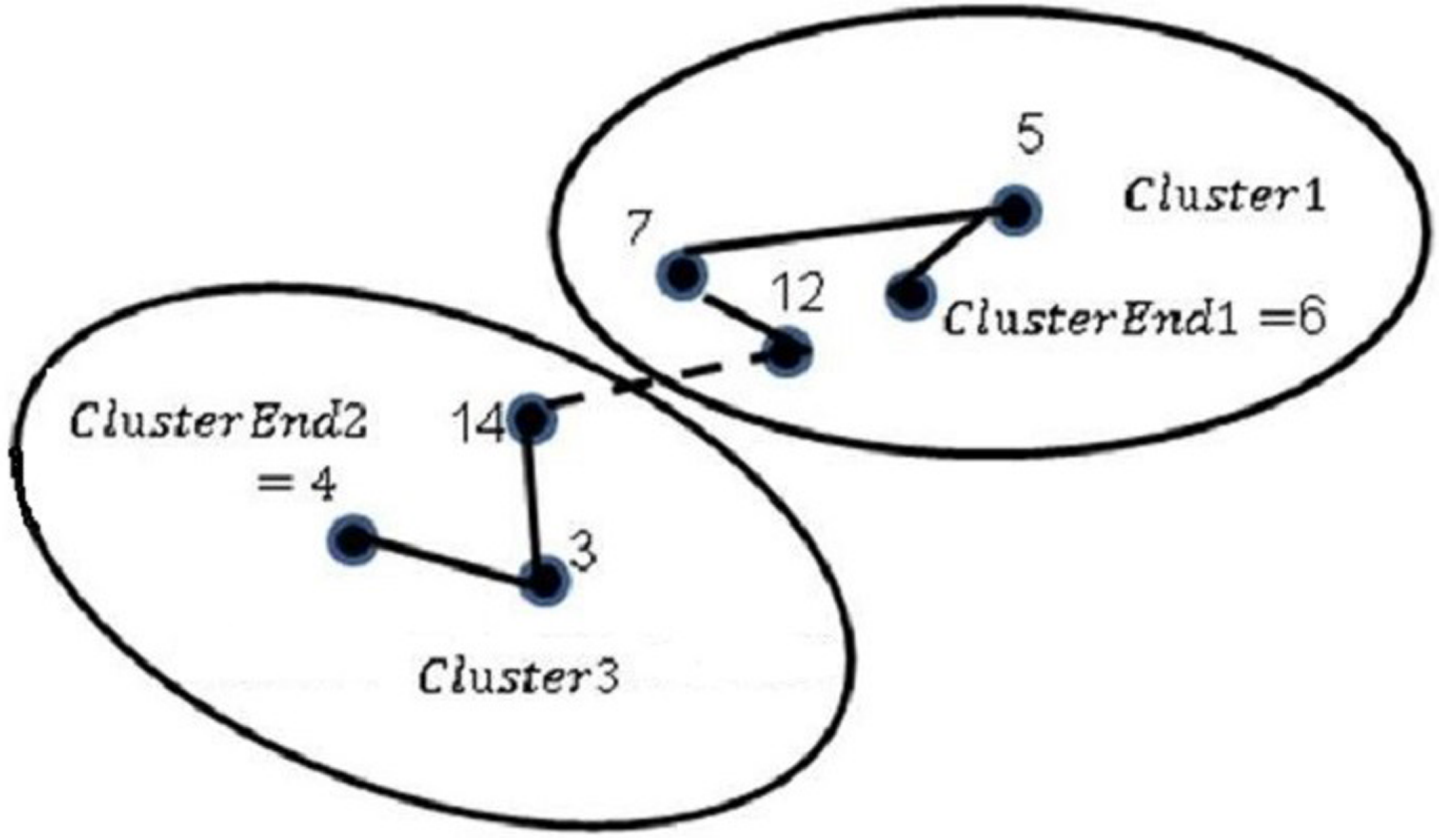
Union of clusters through nearby nodes for MRSILS.

Thus, node 6 is closest to node 12, the direction of the Cluster1 path is 12-6-5-7 and *ClusterEnd1* = 7, and then the nodes near the centroids are joined so that node 12 joins node 14 as shown in (Fig 1). The ClusterEnd1 and ClusterEnd2 nodes remain free until they join the rest of the clusters; In case they were the only clusters, these nodes join to obtain a final route. In case of a greater number of clusters, it is necessary to continue with next step.

12. The next cluster to join is defined by finding the minimum distance between ClusterEnd2 and each of the remaining centroids. For this case, only one cluster is yet to be joined, in such a way that the distance of each node of this final cluster corresponding to cluster 3, with respect to ClusterEnd2, it is calculated, as shown in Table 21.

**Table 21:** Distance ClusterEnd2 to cluster 2 nodes.

Node	Distance to node 14
13	4.80
1	6.52
9	7.29
11	7.10
8	5.96
2	6.02
10	9.60

Table 21 identifies that the cluster 2closest to ClusterEnd2 is 13; and it is named as nodeC3. Subsequently, the node nearest to nodeC3, which is 8 and 10, is identified, to assign the direction to the route, these calculations are in Table 22.

**Table 22:** Distance between 8 and 10 to 13 nodes.

Node	Distance to node 13
8	2.3643
10	5.4507

The closest to the nodeC3 is the node 8, so the sequence of cluster 2 is 13-8-11-9-1-2-10, in addition the last node is called ClusterEnd3, in this case it corresponds to 10.

**Fig 2:**
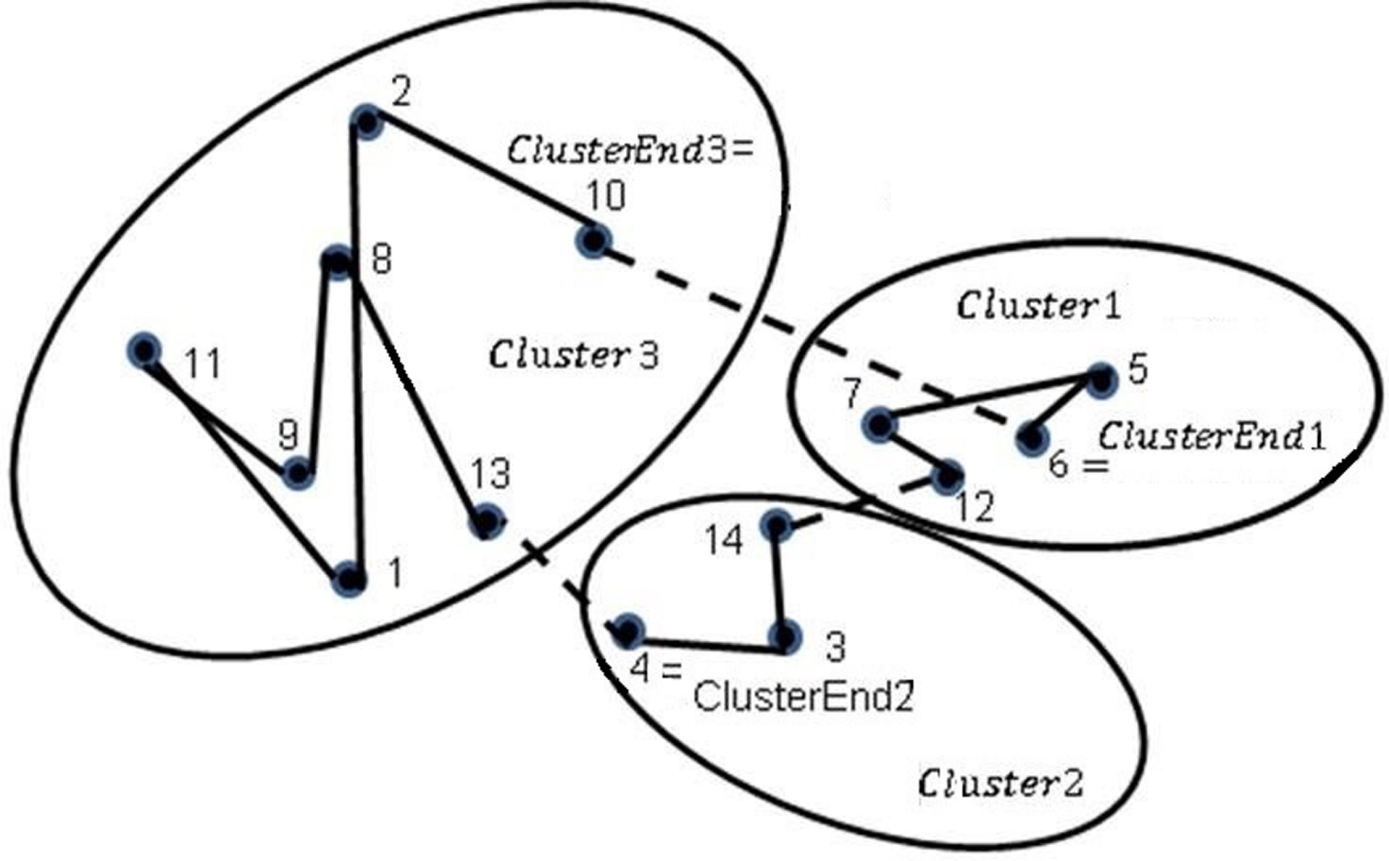
Union of clusters and final route for MRSILS.

13. The previous step is repeated until *k* clusters. Finally, the last node of cluster *k* is joined to ClusterEnd1 to have the final path of the algorithm as shown in (Fig 2).

The final route is obtained: 7-5-6-12-14-3-4-13-8-11-9-1-2-10-7 at a cost of 37.6361. In the next section the results obtained in various instances reported in TSPLIB [50], are compared in both methods Genetic Algorithms and the proposed method described in this section.

## 5 Results

As already mentioned, the objective of this article is to demonstrate that *CTSPMRSILS*, is more efficient than GA when clusters are used in TSP. For comparing them, a GA was programmed with the same parameters of [11], a) Selection Method: Tournament, b) Crossover Rate = 0.9, c) Mutation rate = 0.8, d) Number of generations = 5*n* and e) Number of individuals = 3*n* and *n* is the number of nodes. The 10 instances suggested by the same author were compared in cost and computational time, the last numbers in the name of the instance represent the number of nodes, for example, *rat783* has 783 cities, the distance between the nodes were taken of TSPLIB [50]. Additionally, 30, 50 and 100 runs were used in both methods. The results are shown in Table 23 for the cost and in Table 24 for the time, in both cases, *CTSPMRSILS* obtains better results. It is important to mention that for the case *pcb442* it was not possible to run the GA with 100 runs and for *rat783* it was not feasible 30, 50 or 100 runs. Due to the complexity of the calculations a program was developed in the specialized software MatlabR2015a, and all the examples were solved in a computer with Core Intel Xeon Processor 3.2 GHz—Quad—Memory 8 GB.

**Table 23:** Comparison between CTSPMRSILS and GA, parameter cost, minimum values.

	Cost (Distance)					
	CTSPMRSILS	GA	CTSPMRSILS	GA	CTSPMRSILS	GA
Instance	30 runs	30 runs	50 runs	50 runs	100 runs	100 runs
ei51	491.04	506.07	442.78*	665.85	442.78	486.66
berlyn52	8226.30	8285.09	7785.53	8285.09	7440.13	8040.86
eil76	613.81	663.04	586.94	665.85	599.16	653.71
pr76	113868.00	138224.12	111083.63	137632.47	117142.17	137632.47
kroE100	24986.22	28857.30	23247.92	29774.02	24582.31	28857.29
kroB200	34537.99	47513.69	34523.88	47513.69	33811.15	47513.69
gil262	2857.89	4079.95	2785.60	4079.95	2785.60	4058.39
lin318	49847.67	70550.33	49707.42	70550.33	49407.42	70550.33
pcb442	61465.07	98082.15	61733.09	98082.15	61456.08	
rat783	10463.17		10510.75		10593.16	

*The best results are shaded

**Table 24:** Comparison between CTSPMRSILS and GA, parameter time, minimum values.

	Time (seconds)					
	CTSPMRSILS	GA	CTSPMRSILS	GA	CTSPMRSILS	GA
Instance	30d-min	30d-min	50d-min	50d-min	100d-min	100d-min
ei51	2.3944	26.1599	2.4114	21.5184	2.3354	21.790338
berlyn52	0.6235	15.3622	0.6639	17.127	0.5895	16.9816
eil76	6.8367	74.7216	6.9575	75.044	6.6536	77.5025
pr76	1.5168	73.987037	1.5526	60.969714	1.6365	59.8227
kroE100	13.575	143.9848	13.9632	140.8241	13.386	145.0725
kroB200	18.6667	817.2188	75.50791	861.5537	73.4575	852.7318
gil262	163.469459	1554.1547	158.7468881	1542.3231	152.8723163	1583.0809
lin318	256.4025	2741.5503	63.0805	2841.9702	274.5424	2812.898
pcb442	156.691797	5755.6338	166.6814	6265.4428	149.0371	
rat783	661.92		712.58		705.09	

In addition, 95% confidence intervals and means were carried out to guarantee the certainty of the result, in both indicators minimum cost in Table 25 and computational time in Table 26, *t*-student was used for the mean test, in every case, a test for variances was did before, due to the amount of data the tests of variances and means are based on a normal distribution. *CTSPMRSILS*, represents *μ*_1_ and the GA represents *μ*_2_. In both cases, there is statistical evidence to affirm that the *μ*_1_ is less than *μ*_*2*_, which means that the minimum cost and time are obtained with *CTSPMRSILS*.

**Table 25:** Confidence intervals and p value, cost.

	Cost					
	30 runs		50 runs		100 runs	
	Confidence interval	p-value	Confidence interval	p-value	Confidence interval	p-value
ei51	(-50.60, -17.22)	0.0000	(-59.48, -30.54)	0.0000	(-43.52, -25.18)	0.0000
berlyn52	(-1336, -664)*	0.0000	(-1212, -703)	0.0000	(-1117.1, -747.0)	0.0000
eil76	(-80.24, -54.93)	0.0000	(-92.11, -66.55)	0.0000	(-79.31, -63.21)	0.0000
pr76	(-28595, -20033)	0.0000	(-26384, -20620)	0.0000	(-24328, -20151)	0.0000
kroE100	(-6767, -5044)	0.0000	(-6305, -4984)	0.0000	(-6481, -5509)	0.0000
kroB200	(-14854, -13247)	0.0000	(-14715, -13491)	0.0000	(-14905, -13694)	0.0000
gil262	(-1400.9, -1279.8)	0.0000	(-1413.6, 1308.1)	0.0000	(-1401.1, -1330.5)	0.0000
lin318	(-26517, -23757)	0.0000	(-26937, -24712)	0.0000	(-26052, -24519)	0.0000
pcb442	(-38309, -36287)	0.0000	(-38177, -36699)	0.0000		

*The non-shaded results are those where equal variances are assumed to perform the means test, the shaded are with unequal variances

**Table 26:** Confidence intervals and p-value, time.

	30 runs		50 runs		100 runs	
	Confidence interval	p-value	Confidence Interval	p-value	Confidence interval	p-value
ei51	(-25.887, -25.227)	0.00	(-24.450, 23.838)	0.00	(-24.9907, -24.6556)	0.00
berlyn52	(-26.340, -23.925)*	0.00	(-27.923, -26.453)	0.00	(-27.449, -26.711)	0.00
eil76	(-70.425, -69.402)	0.00	(-70.448, -69.457)	0.00	(-77.870, -76.932)	0.00
pr76	(-72.9670, -72.6858)	0.00	(-70.992, -70.031)	0.00	(-70.202, -69.069)	0.00
kroE100	(-133.414, -131.414)	0.00	(-128.697, -127.451)	0.00	(-133.125, -132.072)	0.00
kroB200	(-818.23, -810.95)	0.00	(-795.74, -785.87)	0.00	(-782.791, -779.615)	0.00
gil262	(-1397.38, -1390.80)	0.00	(-1389.92, -1384.37)	0.000	(-1432.69, -1427.50)	0.00
lin318	(-2537.5, -2484.2)	0.00	(-2833.48, -2809.21)	0.00	(-2586.94, -2564.63)	0.00
pcb442	(-5974.1, -5920.2)	0.00	(-6303.5, -6218.7)	0.00		

*The non-shaded results are those where equal variances are assumed to perform the means test, the shaded are with unequal variances

Also, the best results of the *CTSPMRSILS* were compared with the best-known result reported in the TSLIB [50], the same was done with the results of Piehtrankul [11], see Table 27; in [11] clusters with the *k*-means method and Genetic Algorithms are used. The comparison method was the percentage relative error, being 10.99% in *CTSPMRSILS* against 22.28% obtained with [11]. Which means that the proposed method is better than GA in clusters.

**Table 27:** Relative error, piethtraankul [11] and CTSPMRSILS.

Instance	k-means- Piethrankul [11]	CTSPMRSILS	Best known in TSLIB[50]	Relative error* Piethrankul [11]	Relative error* CTSPMRSILS
ei51	484	442.7835	426	13.62%	3.94%
berlyn52	8416	7440.1273	7542	11.59%	-1.35%
eil76	624	586.938	538	15.99%	9.10%
pr76	125243	111083.63	108159	15.80%	2.70%
kroE100	25918	23247.92	22068	17.45%	5.35%
kroB200	34879	33811.15	29437	18.49%	14.86%
gil262	2801	2785.6	2378	17.79%	17.14%
lin318	51746	49707.4202	42029	23.12%	18.27%
pcb442	63851	61465.0688	50778	25.75%	21.05%
rat783	14370	10463.1679	8806	63.18%	18.82%
			**Average**	**22.28%**	**10.99%**

*The relative error is with respect to the best-know in TSLIB[50]

## 6 Discussion

This article seeks to improve the efficiency of algorithms to solve problems with a larger number of nodes, to achive this goalclustering is used. In this research, computational experiments on 10 different instances of TSPLIB [50] are solved with the intention for comparing two methods: *CTSPMRSILS* and GA when are used in clusters. In this research, computational experiments on 10 different instances of TSPLIB [50] are solved with the intention for comparing two methods: *CTSPMRSILS* and GA when are used in clusters. For made that comparison, a GA was programmed and evaluate in cost and time with *CTSPMRSILS*. Also, some instances found in the literature with clusters and *GA* [11] are compared.

As can be seen in the previous section, the CTSPMRSILS improves the results of the GA when clusters are applied to the TSP. This can be seen in the confidence intervals of both cost and time, since they make inference of the difference that will exist with some confidence between the difference in the results of the compared algorithms, favoring the proposed method. Additionally, when comparing the results obtained by Piethrankul [11] and the proposed method with the best-known found, better results were obtained with the CTSPMRSILS in all instances. Even in the case of *berlyn52* the best-know of TSLIB [50] was improved. Moreover, it can be seen in Table 24, that the best results in 9 of the 10 instances were obtained at 50 runs, so it is suggested in a future work to analyze if the number of runs could be a halt criterion.

## 7 Conclusions

There are a lot of methods to solve the TSP, exact algorithms like branch and cut that are difficult to programming and implement. In the other hand, there are a lot of metaheuristics to deal with the complexity of the problem but any of them do not ensures finding the optimum in polynomial time. For this reason, we presented in our proposal a new algorithm.

Our proposal is a combination of NEH and a modification of the metaheuristic Multi Restart Iterated Local Search MRSILS that are used to solve the TSP with clusters, in the literature there is no one who has used this algorithm to solve the TSP when it is divided into clusters. Phietrankul made a comparison between different algorithms, and GA with cluster was the algorithm that would find the best results (minimum cost). The aim of this article is to demonstrate that the proposed algorithm CTSPMRILS is more efficient than Genetic Algorithms when clusters are used.

We compare CTSPMRSILS with GA with the same parameters of [11] and we get better results with the proposed method. Also, we did the comparison with the results published by Piehthrankul and we obtained better results in all the instances tested. We conclude that method proposed in this article is a viable candidate to solve problems as required by manufacturing companies and obtain better results in cost and time compare with GA.

In addition, the following recommendations are proposed for future research:

1. The clustering is perfectible so that different methods could be for optimizing the allocation of the nodes to the different clusters.
2. It is feasible to consider the combination of the MRSILS with some Metaheuristic different from the NEH in the search of better results.
3. It could also be applied as a halt criterion for predetermined runs in the MRSILS.
4. One more recommendation may focus on proposing a different method for joining clusters, after metaheuristics give a result.
